# The Microbiome of Endophytic, Wood Colonizing Bacteria from Pine Trees as Affected by Pine Wilt Disease

**DOI:** 10.1038/s41598-017-04141-6

**Published:** 2017-06-23

**Authors:** Diogo Neves Proença, Romeu Francisco, Susanne Kublik, Anne Schöler, Gisle Vestergaard, Michael Schloter, Paula V. Morais

**Affiliations:** 1 CEMMPRE, University of Coimbra, Coimbra, Portugal; 2 Research Unit Environmental Genomics, Helmholtz Zentrum München, Munich, Germany; 3 Department of Life Sciences, University of Coimbra, Coimbra, Portugal

## Abstract

Pine wilt disease (PWD) is a devastating forest disease present worldwide. In this study we analyzed the effects of the invasion of the pinewood nematode *Bursaphelenchus xylophilus*, the major pathogen causing PWD, on the endophytic microbiome of adult *P*. *pinaster* trees. Wood samples from trees with different degrees of PWD disease were collected at two sites (A and M) in Portugal. Endophytic bacteria were characterized based on directly extracted DNA by fingerprinting and barcoding using the 16S rRNA gene as marker. Furthermore, cultivation-based approaches were used to obtain isolates of the major taxa to study their ecophysiology. The endophytic microbiome from *P*. *pinaster* trees differed significantly between the two sampling sites. Main bacterial OTUs belonged to the *Proteobacteria* (39% (site M) - 97% (site A)), and *Firmicutes* (0.70% (site A) - 44% (site M)). However, consequences of the invasion with the pathogen were comparable. Interestingly diversity of wood endophytic bacteria increased with the severity of the diseases, with highest diversity levels observed in in the most affected trees. Our results suggest that in the first stages of the disease, the defence mechanisms of plants are repressed by the pathogen, resulting in a colonization of the wood interior by soil microorganisms.

## Introduction

Native to North America (the USA and Canada), pine wilt disease (PWD) is one of the most devastating forest diseases in the world. The disease has spread to Asia and to Europe, mainly affecting Japan, China, Korea, Taiwan, Portugal and Spain^1^. In Portugal, PWD was reported first in 1999 by Mota and coworkers^2^, in an area south of the Tagus river; after 2008, PWD has spread to the center and north part of the country^3^. Portuguese forests are mainly composed of several species of pine, eucalyptus and oak trees. The most abundant species of pine, *P*. *pinaster*, making up 62.5% of total trees, is a PWD-susceptible tree species^3^.

*Bursaphelenchus xylophilus*, the pinewood nematode, has been considered for a long time to be the only known causal agent of the disease^4^. Once having entered the plant inoculated by the insect vector when feeding on young shoots, the nematode destroys the vascular system of plants^5^. The nematodes can reproduce quickly in the sapwood under favourable conditions within susceptible pine species, causing wilting and death, sometimes in only a few weeks. In order to make phytosanitary decisions, a classification based on the aspect of the crown and the colour of the leaves was created^6^. This classification system includes six classes from trees without symptoms (s0) to trees without leaves (sV).

A relationship of mutuality has been suggested between bacteria and *B*. *xylophilus* ^7^ in the development of the disease. The origin of these bacteria, their functional traits as well as which plant compartments they colonize is still under discussion^6, 8–12^. In this study we addressed the question how the diversity of endophytic bacteria colonizing the wood interior changes as a result of PWD.

Endophytic bacteria have been shown to provide different services to the plants. Besides acting as plant growth promoters^13^, they can exhibit strong anti-fungal activity, antagonise bacterial pathogens and control plant parasitic nematodes^14–17^. Consequently changes in the microbiome structure of endophytes might have pronnounced effects for the plant phenotype. Several studies have confirmed the presence of endophytic microbes as part of the *Pinus* spp. holobiont. In Scots pine, *P*. *sylvestris*, the major genera of endophytic bacteria found were *Methylobacterium*, *Pseudomonas* ^18–20^, *Bacillus* and *Paenibacillus* ^21^. *Paenibacillus* and *Bacillus* were also found as endophytes of *P*. *contorta* ^22, 23^, while the genera *Acetobacter* and *Gluconacetobacter* were part of the foliar endophytic community of *P*. *flexilis* ^24^.

Taking in consideration that the pinewood nematode produces effector proteins, as cellulases and peroxiredoxin^4^, that modulate the plant response and might reduce the plant defenses, we postulated that the infection with *B*. *xylophilus* of pine trees will change the structure of bacterial endophytes. We hypothesize that the diversity of endophytes will increase mainly in heavily infected trees, due to reduced plant defense mechanisms against entering the plant interior. This might overall accelerate the course of the disease. To study these hypotheses, we extracted microbes from the woody part of *P*. *pinaster* trees from two regions in Portugal. In both regions, trees with different degree of severity of the disease were present. We used molecular fingerprinting and barcoding methods to describe bacterial diversity based on extracted community DNA and amplified 16S rRNA genes, as well as isolation based approaches to assess the phenotypes of major (cultivable) wood colonizing endophytes.

## Results

### Bacterial community fingerprinting

The overall diversity of wood colonizing bacterial endophytes was assessed from 43 *P*. *pinaster* trees, with differing severity of PWD as well as unaffected healthy trees from two regions in Portugal (Table S1). The DNA used was extracted directly from a microbial fraction, obtained from the core of the woody material obtained from the trunk respectively crown, and amplified 16S rRNA genes were subjected to DGGE based fingerprinting.

**Figure 1:**
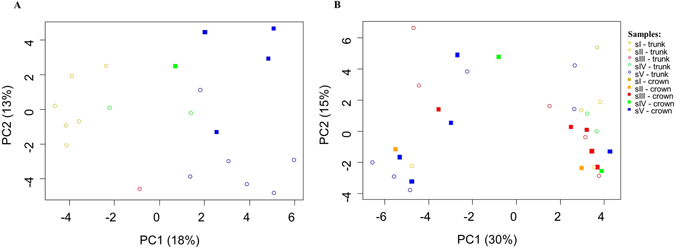
Principal component analysis (PCA) of DGGE fingerprinting of endophytic, wood colonizing bacterial communities from all pine wood tree samples from the two sampling sites obtained after PCR amplification of 16S rRNA gene fragments. The samples grouped according to the different symptomatic physiological classes in Malhada site (**A**); no clear clustering was observed in Avô site (**B**).

Overall, between 30 to 44 bands were obtained per sample, when the bacterial community fingerprints were analyzed (data not shown). After excluding bands related to chloroplast sequences, no significant differences in diversity according to Shannon-Weaver and Simpson 1-D indexes were found when both sites, Avô and Malhada, were compared (H′ = 2.86 and 3.45; D = 0.94 and 0.96, respectively); at both sites for diseased trees of the highest symptom class (sV), a higher diversity was visible (H′ = 2.92 and 3.27; D = 0.94 and 0.96) compared to healthy trees (s0) (H′ = 2.71 and 3.13; D = 0.93 and 0.95) (Table S2). Comparing the presence and absence of individual bands, the clearest differences for bacterial communities were obtained when comparing trees from both sites (paired samples *t*-test, *t*_43df_ = 5.50, *p* < 0.001) (Fig. 1). Whereas at site A no clear clustering was observed according to the disease symptoms, at site M, mainly the trees with the highest degree of disease outbreak differed from the other trees. Overall DGGE pattern did not change when samples from crown and trunk from the same tree were compared.

### Bacterial community barcoding

A total of 2,791,770 high quality reads were generated by sequencing 16S rRNA gene based amplicons derived from community DNA from 12 samples covering trees of six different symptomatic physiological classes from the two sampling areas (Table S3). The total number of reads ranged from 55,806 (A-sI) to 208,182 (A-sIV). After filtering and removal of 16S rRNA gene sequences derived from chloroplasts, reads ranged from 24,069 to 176,403 for M-s0 and A-sV, respectively. Thus, for further analysis all reads from all sequenced samples were subsampled to 24,069 reads. The difference observed in read numbers for the individual samples were mainly a result of sequences which were identified as chloroplasts from Pine trees (0.3–62%), which were obviously co-extracted during the preparation procedure. However, even after subsampling, the analysis of rarefaction curves showed for all samples sufficient sequencing depth to assess OTU diversity of early classes of disease (s0-sII) at a level of 97% similarity, while a large increase of OTU diversity, at classes sIV and sV, was observed mainly from trees at site M (Figure S2). Representative sequences from each OTU were phylogenetically classified using the Greengenes database. The number of detected OTUs ranged from 454 (A-sI) to 2,260 (M-sIV).

**Figure 2:**
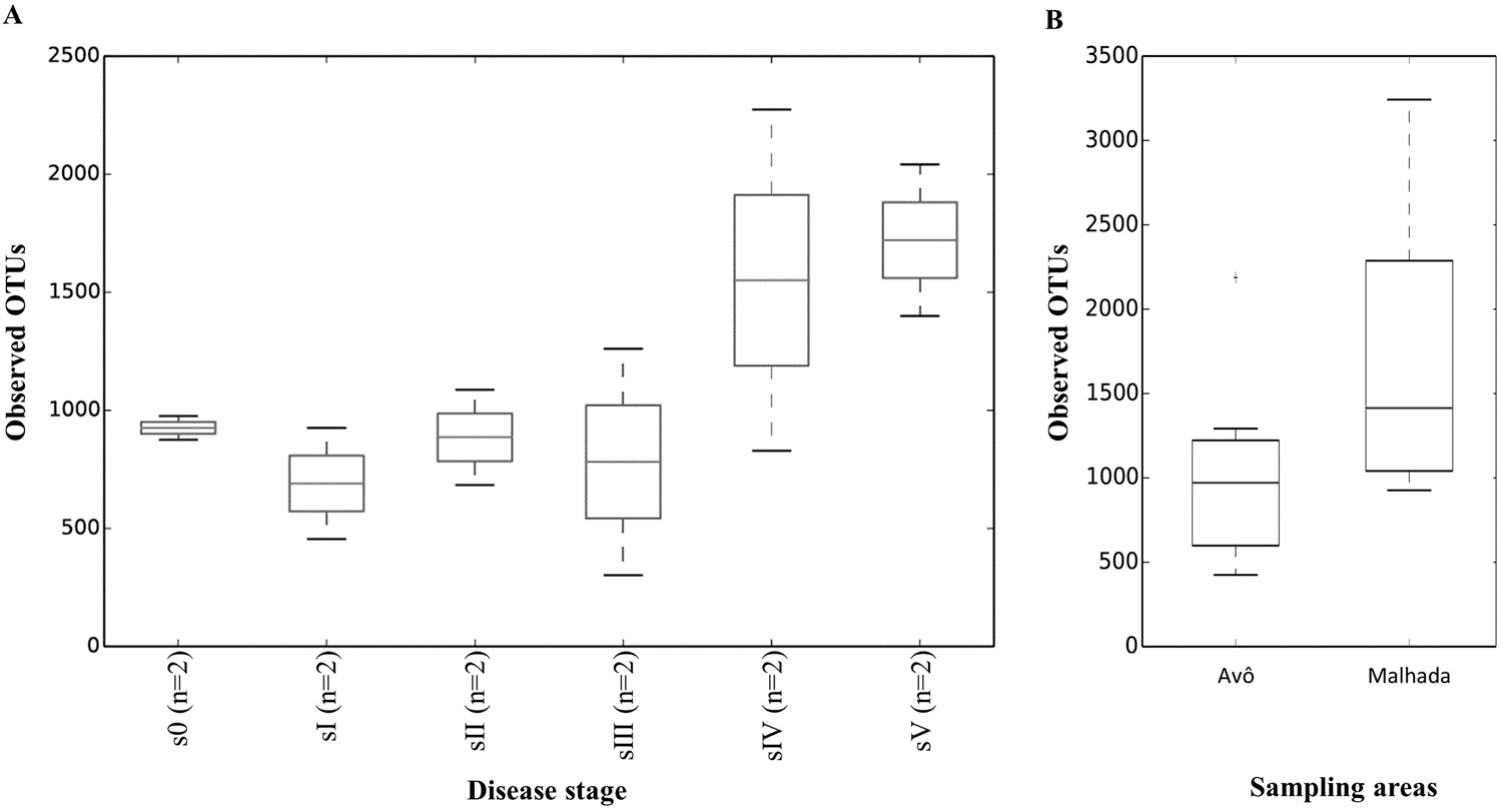
α-Diversity analysis of endophytic, wood colonizing bacterial communities from pine trees of six different symptomatic classes from the two sampling areas based on sequencing of PCR amplified 16S rRNA gene fragments (Chao1). (**A**) Comparison of different symptomatic stages (**B**) Comparison of sites.

Interestingly, the highest diversity was found in the wood samples of the most diseased trees both for site A and site M, which confirms the data of the fingerprint analysis of the individual samples. Overall, diversity of OTUs was higher at site M compared to site A (H′ = 5.72 and 4.29; D = 0.97 and 0.95, respectively) but no statistical differences were found between these two sites, (Table S2). Also the alpha diversity measured based on Chao1 (richness) indicated higher diversity at site M compared to site A (Fig. 2). Chao1 based analysis also indicated that trees in different symptomatic stages presented different endophytic community richness, with lower diversity in the first stages of the disease and higher diversity at later stages (sIV and sV).

**Figure 3:**
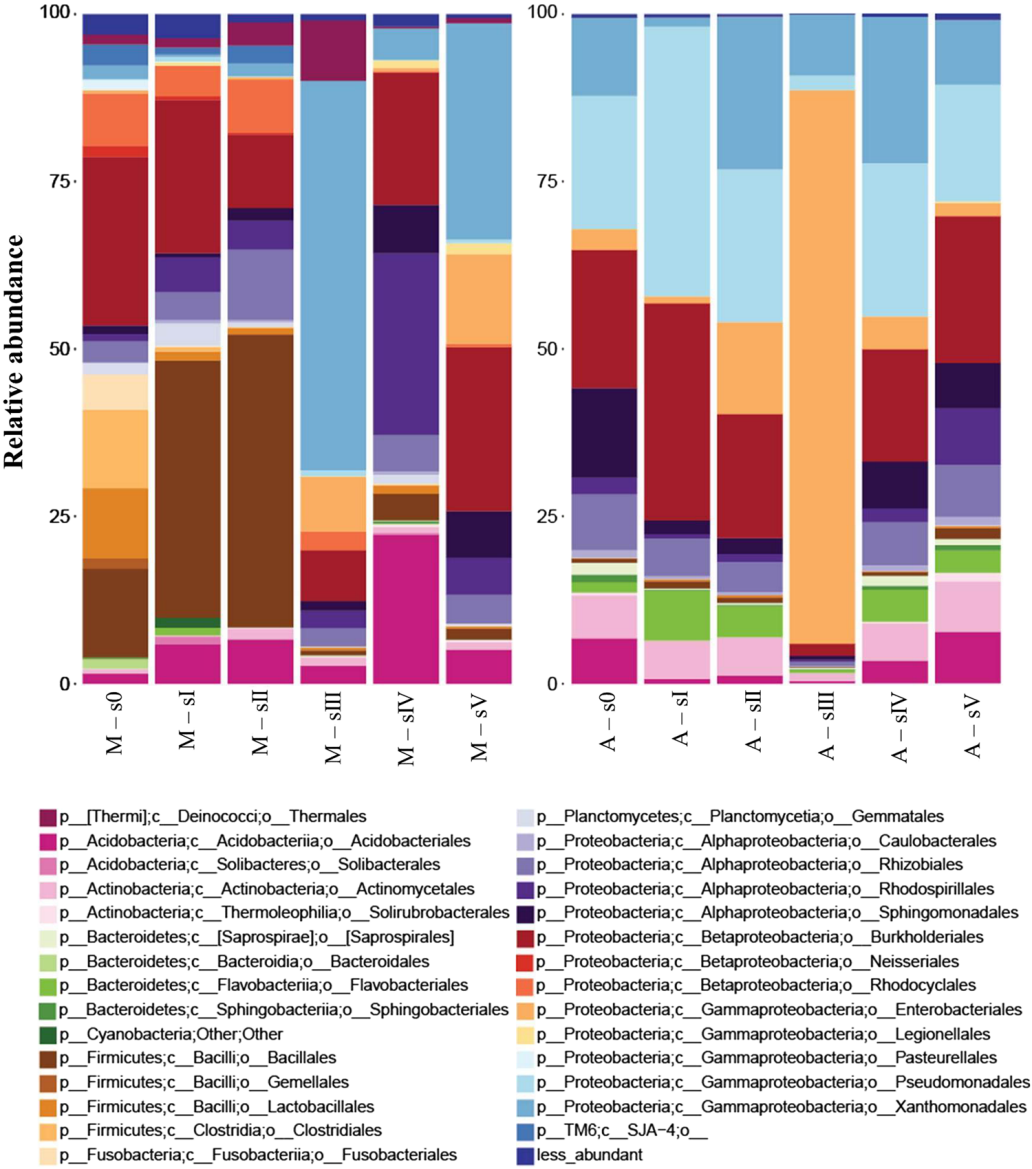
Diversity of endophytic, wood colonizing bacterial communities at the level of classes from pine trees of six different symptomatic classes from the two sampling areas (A and M) based on sequencing of PCR amplified 16S rRNA gene fragments. Only bacterial classes higher in abundance than 0.5% were included.

Like expected from the fingerprint analysis, our results demonstrated that the composition of the endophytic communities from pine trees differed in response to the investigated site but also to the symptomatic stages (p < 0.001) (Fig. 3). *Proteobacteria*, varying between 39% (M-sII) and 97% (A-sIII), and *Firmicutes* ranging from 0.70% (A-sIV) and 44% (M-sII), were the predominant endophyte bacterial phyla in the pine trees at both sites, followed by *Actinobacteria*, *Bacteroidetes*, *Acidobacteria* at site A, and *Fusobacteria*, *Acidobacteria* and *Thermi* at site M. Members of *Proteobacteria* included mainly *Gammaproteobacteria* (1% (M-sI) - 94% (A-sIII)), *Alphaproteobacteria* (5.71% (M-s0) - 40% (M-s4)) and *Betaproteobacteria* (10% (M-sIII) - 34% (M-s0)). *Bacilli* (<1% (A-sIV) - 44% (M-sII)) and *Clostridia* (<1% (A-sIII - 11% (M-s0)) were the predominant *Firmicutes*.

**Figure 4:**
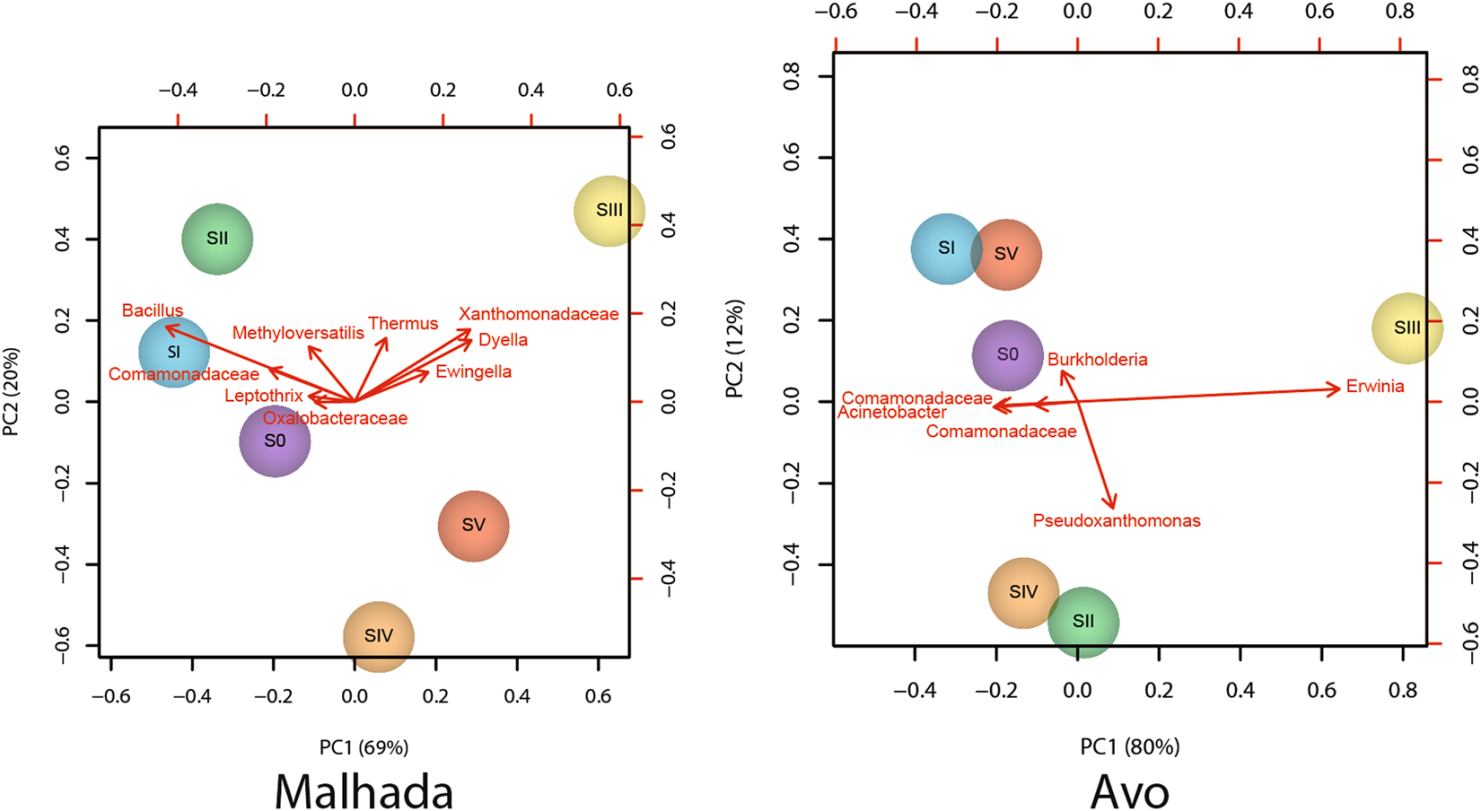
Principal component of endophytic, wood colonizing bacterial communities at different phylogenetic levels from pine trees of six different symptomatic classes from the two sampling areas (**A**: Malhada **B**: Avo) based on sequencing of PCR amplified 16S rRNA gene fragments.

At site M healthy (s0-sII) trees harbored a large number of OTUs which could be related to *Bacillus* and *Streptococcus*; this number was significantly reduced at later symptom stages (sIII-sV). At site A, however, *Bacilli* related OTUs only played a minor role and did not change in response to the different symptom stages. *Xanthomonadaceae*, in the case of site M and *Enterobacteriaceae* at site A; were the dominant endophytic bacterial group in the trees of symptomatic stage III, which had the most distinct microbiome according to Chao1 analysis at both sites (67% at M - 94% at A), with high abundance of *Serratia* and especially *Erwinia* found at site A (8% at M - 82% at A) while *Dyella* and *Pseudoxanthomonas* were highly enriched at site M (57% M - 9% A). These results were also confirmed by a PCA analysis (Fig. 4).

### Isolation and characterization of endophytic bacteria

Colony-Forming Units per gram of wood (CFU/g) varied from 1.5 × 10^3^ ± 7.0 × 10^2^ to 1.5 × 10^6^ ± 6.3 × 10^5^ in area M and from 7.1 × 10^1^ ± 3.7 × 10^1^ to 2.3 × 10^7^ ± 7.0 × 10^6^ in area A. In general, the number of bacteria was higher in site A than in site M as found by community barcoding. Furthermore, symptomatic trees showed more CFU per gram of wood than healthy trees or trees with low level symptoms. In 11 trees of area M and 7 trees of area A, no bacterial isolates were obtained, most of them in healthy condition (data not shown).

In total, 313 endophytic bacterial isolates were grouped in 277 RAPD group types (data not shown). Overall, 16S rRNA genes from 144 isolates and 133 isolates from site M and A, respectively, were sequenced, representing 162 bacterial isolates (M) and 151 bacterial isolates (A). As expected, not all of the taxa detected by sequencing were cultivatable under the conditions used.

Diversity of isolates was comparable at site A and site M (H′ = 2.90 and 2.92; D = 0.91 and 0.92, respectively). As found by barcoding, the highest diversity was found in the samples of the most diseased trees in both sites (Table S2). In accordance to the fingerprint and barcoding data, redundancy analysis (RDA) indicated that the majority of the trees clustered according to the sampling areas and according to the symptomatic stage of the trees inside each sampling site (data not shown). Also one-way ANOVA showed statistical differences between healthy (s0-sIII) and symptomatic trees (sV) in both areas (p < 0.05, respectively), when isolates were analyzed on the genus level (Figure S1). Differences were not observed between symptomatic trees (sV) of site A and site M (*p* > 0.05).

**Figure 5:**
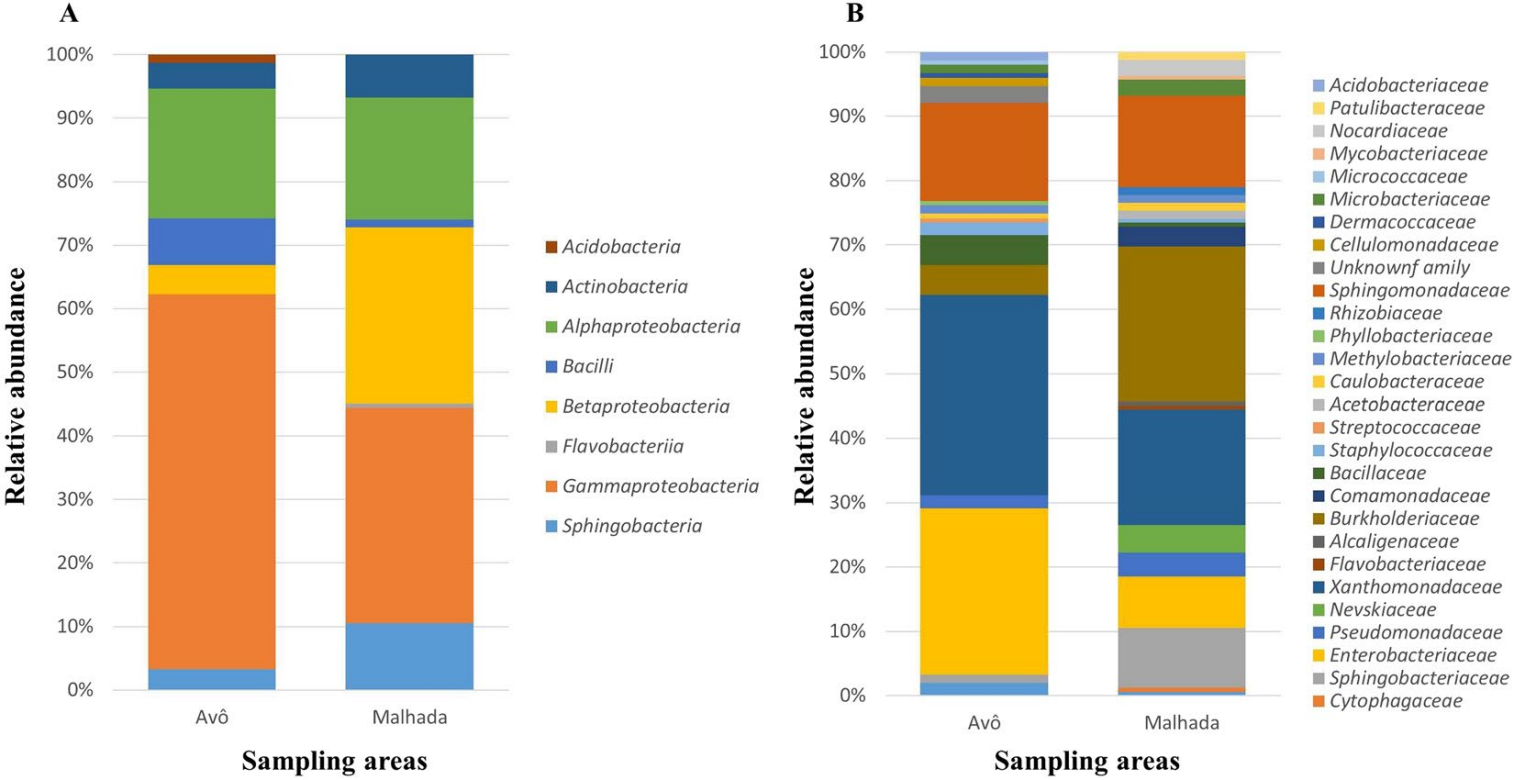
Phylogenetic characterization of endophytic, wood colonizing bacteria from pine trees of the two study sites (A and M) obtained by cultivation based approaches at the level of phyla (**A**) and families (**B**).

*Proteobacteria* were the most abundant isolates at both sites (80–85%), independently of the degree of PWD (Fig. 5). Whereas at site A 59% of the isolates were classified as *Gammaproteobacteria* (*Enterobacteriaceae*, *Pseudomonadaceae*, *Xantomonadaceae*), followed by *Alphaproteobacteria* (20%) and *Betaproteobacteria* (4%), at site M *Gammaproteobacteria* were less abundant (35%) but the abundance of *Betaproteobacteria* was much higher (28%); the number of isolates for *Alphaproteobacteria* was comparable between both sites. These differences in the abundance of *Gammaproteobacteria* were mainly related to different numbers of isolates from *Xanthomonadaceae* (31% and 17% of the isolates for site A and M, respectively). For *Betaproteobacteria*, diverging numbers of *Burkholderiaceae* isolates were responsible for the differences observed for site A (4%) and M (24% with the additional families *Commomonadaceae* and *Alcaligenaceae*). Overall, besides *Proteobacteria* also *Acidobacteria*, *Actinobacteria*, *Bacilli*, *Flavobacteriia* and *Sphingobacteria* were isolated but with low frequency.

At site M, the genera *Pseudoxanthomonas*, *Ewingella* and *Brevundimonas* were the most abundant at sIII, *Burkholderia* and *Sphingomonas* at sIV, and *Ralstonia*, *Sphingomonas*, and *Pseudoxanthomonas* at sV. Moreover, *Variovorax* and *Sphingomonas* were common in all the symptomatic classes. While the most abundant genera at site A were *Streptococcus*, *Staphylococcus* in s0, isolates belonging to the genera *Erwinia* in sI, *Pseudoxanthomonas*, *Erwinia*, *Methylobacterium* and *Pantoea* in sII, *Pseudoxanthomonas*, *Erwinia*, *Serratia*, *Klebsiella* in sIII, *Pseudoxanthomonas*, *Enterobacter* and *Pantoea* in sIV, and *Sphingomonas*, *Pseudoxanthomonas* and *Burkholderia* in sV.

One hundred and eighty-five isolates representatives of the OTU detected with more than 1% abundance were phenotypically characterized. Interesting, most of the 29 strains of *Erwinia*, which genus includes 81% of the OTU of sIII trees at site A, were positive for all tested characteristics except chitin degradation. In site M, six strains of *Dyella* (28.2% of the OTU in sIII), were positive for lipases, proteases, siderophores and zinc solubilization (Table S4).

Isolates from site A tend to have more proteolytic and cellulolytic activity when isolated from trees in symptomatic sV, whereas at site M, no clear pattern was visible and the functional activities of the isolates from trees in the different symptomatic stages were similar (Figure S3).

## Discussion

Endophytic bacteria exist within the living tissues of probably all plants, but compared with herbaceous plants, remarkably little is known about their ecology in trees. In this study, we assessed for the first time the diversity of endophytic microbial community of Portuguese *P*. *pinaster* trees, by cultivation and molecular methods, focusing on trees infected and non-infected with *B*. *xylophilus*.

All sampled trees were colonized by a diverse endophytic community, although in some cases no isolates were obtained and the presence of these microbes was only described by 16S rRNA gene barcoding. Interestingly when considering the composition of the endophytic microbiome, pine trees grouped by sampling areas mainly. The main components of the community were *Proteobacteria* of the class *Gammaproteobacteria* and, depending on the sampling area, also *Firmicutes*. Six families were the most abundant found in all symptomatic stages from both sampling areas – *Bacillaceae*, *Burkholderiaceae*, *Enterobacteriaceae*, *Comamonadaceae*, *Sphingomonadaceae* and *Xanthomonadaceae*. However, the relative abundance of bacterial families differed among the two sampling areas. These pronounced differences between the two sites that are geographically close, might be due to differences in environmental conditions, probably related to water availability, pH and soil composition^25^.

As expected, the pathogen impacted the microbiome structure of wood colonizing endophytes. Whereas at early stages of the disease only slight shifts in the bacterial diversity were observed, from sIII on drastic shifts were visible. Overall, an increase in diversity with more severe symptomatic stage was visible. This may be a result of a loss in the ability of the plants to control microbial invasion of the plant interior from outside, or of a loss in the control of the growth of endophytes as a result of the disease. An increase in the endophytic microbial diversity related with the decline of the tree was previously also observed when studying the endophytic fungi of pine trees^26^. The sudden modification of the microbial community of the trees at sIII might be related to the fact, that mostly at this stage the pathogen is detected in higher numbers for the first time after tree infection at the trunk level^27^, which could lead to significant reduction of plant immunity as well as the creating new conditions inside the tree^28^. However, it is also likely that bacteria which are carried by or able to adhere to the pathogen at this stage are present in high abundance simply as a result of the increased number of *B*. *xylophilus*. This assumption is confirmed by the observation that mainly an increase in OTUs linked to *Xanthomonadaceae* and *Enterobacteriaceae*, including *Dyella* and *Ewingella* strains, respectively, which were previously shown to be carried by with the pathogen^12^, increased in number up to 58% of the community at sIII was observed at site M. A phylogenetic analysis of the sequences obtained in this study, which the strains described previously as carried by the nematode^4^ confirmed this assumption (Figure S4). At site A comparable pattern was observed. Here OTUs related to *Erwinia*, again a previously described species associated to the pathogen^12^, increased at sIII. Interestingly the relative abundance of typical plant endophytes for example OTUs related to *Bacillus*, *Comamonadaceae* or *Methyloversatilis* at both sites decreased at the same time.

Although some comparable developments were seen at both sites, the present study indicated mainly at later stages of the disease site specific effects. At site A, the microbiome at symptomatic stage sV resembles community composition similar to early stages of the disease which was not the case in site M. Thus it is quite likely that both the original composition of the microbiome as well as site specific conditions determine the course of the disease in terms of microbial community composition.

Interestingly none of the ten most important plant pathogenic bacteria^29^ were isolated or detected by 16S rRNA gene sequencing in this study, and a pathogenic role for the endophytic community was previously disregarded. However, it is quite likely that most of the new endophytes are capable to act as saprotrophs and degrade important structural elements of plants. This assumption is confirmed by the analysis of the phenotype of the isolates mainly at site A, where higher symptomatic stages lead to an increase in strains with proteolytic and cellulolytic activity. Cellulases (β-1,4-endoglucanses) were also described in the genome and in the microbiome of *B*. *xylophilus* and may help the nematode in the invading the plant tissue^30, 31^. However, for root associated microbes cellulolytic activities have been described as a part of their ability to enter the root^32^. In our study typical soil borne microbes like *Chitinophaga* and *Ewingella* have been isolated carrying those functional traits. Thus it stays unclear if the increase in cellulolytic activities is more related to microbes introduced by the nematode, or microbes that entered the plant interior via the soil.

Overall the presented data gives clear evidence for significant shifts both in the structure and the function of endophytic bacteria with ongoing disease development, which might lead to an additional decline of the tree fitness. Such data might be of great importance when mitigation strategies are developed for example by introducing biocontrol active antagonist like recognized nematicidal bacteria^33^ into the wood material. If such approaches are successful needs to be studied in future experiments.

## Experimental Procedures

### Field sites and sample collection

Samples were collected from Malhada Velha, Arganil (M) and Avô, Oliveira do Hospital (A)^6^. These are two different areas affected by PWD since 2008 from Coimbra District, Central Portugal. The areas include mainly *P*. *pinaster*, with a sparse number of *P*. *radiata*, *Quercus faginea* and *Eucalyptus globulus* trees (Table S1). The sampled trees were classified into six different PWD symptom classes.

Sampling was performed from April to August 2009 according to Proença *et al*.^6^. Briefly, each sample consisted of pinewood cross-sections from cut trees or wood obtained by drilling a 5 mm diameter hole to a depth of 10 to 15 cm with a sterilized hand brace drill (Haglof, Mora, Sweden). Forty-seven trees were sampled at the trunk (1.5 m height). For those pines cut for phytosanitary purposes, an additional sample was obtained from the crown of the tree. From area M, 18 *P*. *pinaster* trees were sampled. From area A, 25 *P*. *pinaster* trees were sampled (Table S1). Sampled trees had an average trunk diameter at breast height of 19.9 ± 8.03 cm in Malhada and 24.7 ± 10.8 cm in Avô. All samples were stored at 4 °C after sampling and further processed as described below latest one day after sampling.

The presence of nematodes was screened in all sampled trees using modified Baermann funnels^34^. *B*. *xylophilus* was detected in three symptomatic *P*. *pinaster* trees area M (M24, M47, M67) and 14 symptomatic *P*. *pinaster* trees from area A (A12, A25, A36, A37, A41, A52, A60, A72, A88, A96, A103, AB20, AB23, AB29). *B*. *xylophilus* was not detected in healthy trees.

The bark and sapwood of each sample were removed under sterile conditions and the wood cut in pieces of the size of about 1 × 0.5 × 0.5 cm. Microbes were released from the wood material by shaking 5 g of wood pieces at 25 °C, for 2 h in 0.9% NaCl. The resulting suspensions were either stored at −20 °C for molecular analysis or directly processed for isolation based approaches.

### Extraction of community DNA

Total DNA of microbes extracted from each tree wood sample was obtained, following the method of Nielsen *et al*.^35^ with an extra incubation step with 1% CTAB (cetyl trimethyl ammonium bromide)/0.7 M NaCl at 65 °C for 20 min.

### Fingerprinting of endophytic bacteria

The extracted community DNA was used as template for fingerprinting of the 16S rRNA gene from Bacteria using denaturating gradient gel electrophoresis (DGGE). The 16S rRNA amplicons were generated using primer pairs 27 F and 1525R^36^ for the first PCR. The obtained amplicons were subsequently used as DNA template for a Nested-PCR by using primer pair 341F-GC clamp and 907R^37^.

Fingerprints were obtained by DGGE analysis using a DCode™ Universal Mutation Detection System (Bio-Rad Laboratories, Hercules, California, USA). PCR samples were loaded into 8% polyacrylamide gels with a denaturing gradient ranging from 30–70% (100% denaturant is defined as 7 M urea and 40% formamide). Gels were run at 70 V, for 17 h, at 60 °C and stained with ethidium bromide.

### Barcoding of endophytic bacteria

The extracted community DNA was further used as template for 16S rRNA amplicon sequencing using MiSeq Reagent Kit v3 (Illumina, San Diego, USA). Therefore the extracted community DNA of each symptomatic state from each sampling area was pooled together, resulting in 12 samples (six classes, from two sampling areas; M-s0 through M-sV; A-s0 through A-sV) and amplified using primers S-D-Bact-0008-a-S-16 and S-D-Bact-0343-a-A-15^38^. Amplicons were verified for their correct size by gel electrophoresis; bands with the expected size were excised and purified by PCR Purification Kit (Macherey-Nagel, Düren, Germany). Each PCR was performed in triplicates, which were pooled and analyzed by Bioanalyzer DNA 7500 Chip (Agilent, Santa Clara, USA). DNA quantification was performed by Picogreen (Thermo Fisher, Waltham, USA). Sequencing was performed using the paired end methods on a Illumina MiSeq (Illumina, Berlin, Germany).

Sequences were trimmed and merged using Adapterremoval^39^ using default values, except ambiguous bases at 5′/3′ termini were trimmed and bases at 5′/3′ termini with quality scores equal or less than 15 were trimmed. Contaminating PhiX sequences were removed using DeconSeq v.0.4.3^40^. Chimeric sequences and plastid sequences were removed by using QIIME v.1.9.1^41^. OTU picking was done using Open-reference OTU picking against the 97% similarity clustering of Greengenes as of May 2013^42^. Basic diversity analysis was likewise done using Qiime v.1.9.1 at a subsampling of 24,069 reads. Stacked barplots of the taxonomical distribution, filtering away all OTUs with less than 1% of reads per sample, was done using R v.3.2.4 (http://r-project.org). The PCA biplot was done on Hellinger transformed data filtering away all OTUs with less than 5% of reads per sample, also using R v.3.2.4 (http://r-project.org). The following R packages were used: vegan^43^, gplots, gplots2, sciplot, plyr, MASS and gridExtra.

### Isolation and phylotyping of endophytic bacteria

To isolate bacteria from the woody part of pine trees, dilutions of the suspensions obtained as described above were plated on R2A agar. Plates were incubated up to five days, in the dark, at 25 °C. The number of colonies per plate was counted using a stereomicroscope, and the number of colonies per gram of sampled wood was assessed. Colonies from each tree sample with different morphologies were randomly isolated, and preserved in LB medium (Difco Laboratories, Detroit, USA) with 15% glycerol stocks at −80 °C after sub-cultivation and purification.

DNA from each bacterial isolate was extracted according to Nielsen *et al*.^35^ and isolates were grouped by RAPD typing. RAPD fragments were amplified by PCR, using primer OPA-03 as previously described^6^. DNA profiles for isolates were grouped on basis of visual similarities of the fragments analyzed by electrophoresis in a 2% agarose gel stained with ethidium bromide. Representative strains from each RAPD group were phylogenetically identified by 16S rRNA gene sequencing using primers 27 F and 1525 R as described^36^.

All obtained sequences from bacterial isolates were compared with sequences available in the EMBL/GenBank database using BLASTN network services^44^ and with sequences available on the Eztaxon-e server (http://eztaxon-e.ezbiocloud.net/)^45^. Sequences were aligned within the SINA alignment service^46^. Sequences were also checked for chimeric artefacts by using Mallard software^47^. Sequences were included in 16S rRNA-based Living Tree Project (LTP) release 115 database (http://www.arb-silva.de/projects/living-tree/) by parsimony implemented in the ARB software package version 5.5^48^. Phylogenetic dendrograms of sequences from this study and closest reference sequences were constructed by the Randomized Axelerated Maximum Likelihood (RAxML) method with GTRGAMMA model^49^ included in the ARB software^48^.

The phenotype of strains belonging to the genera which were also detected by sequencing of extracted community DNA (>1%) was analyzed focusing on the solubilization of phosphate and zinc, production of siderophores; and hydrolyzing activities including protease (casein), lipase (Tween 20, 40, 60 and 80), cellulase and chitinase activities^50^.

### Statistical analysis

PAST 2.08 software^51^ was used to calculate the Shannon–Weaver index (*H*′) and Simpson indices, one-way ANOVA, paired samples *t*-test and similarity percentage (SIMPER). Indices were calculated for isolates based on number of strains belonging to each phylotype (genera) and for DGGE profiles were based on the presence of each DGGE band.

Based on the results of OTUs obtained by sequencing of the obtained amplicons, alpha diversity was calculated, including Chao1 (QIIME package), as well as Shannon and the Simpson indices (PAST 2.08 software). To estimate Beta diversity, Bray-Curtis dissimilarity matrix were used to examine the similarity of the membership and structure found in the various samples.

Redundancy analysis (RDA) was performed in order to reveal relationships between pine trees, bacterial species and the environmental variables (two sampling areas, PWD symptom class^6^ and presence of *B*. *xylophilus*) by using the software package CANOCO (version 4.5.1). RDA was accompanied by Monte Carlo permutation tests to evaluate the statistical significance of the effects of the explanatory variables on the species composition of the samples^52^.

PCoA based on OTUs was performed in order to understand the pine’s endophytic microbiomes from two sampling areas belonging to six PWD symptom classes.

### Nucleotide sequence accession numbers

The 16S rRNA gene sequences of the isolates, have been deposited in the NCBI Genbank database under the accession numbers according KJ654634 - KJ654916, KT730168 and KT730169. The sequences obtained from the amplification of the 16S rRNA gene using the extracted community DNA as template have been deposited GenBank under the SRA Accession number SRP077519.

## Electronic supplementary material

Supplement
