# Supplementary material for: The Microbiome of Endophytic, Wood Colonizing Bacteria from Pine Trees as Affected by Pine Wilt Disease
Source: Sci Rep. 2017 Jun 23;7:4205. doi: 10.1038/s41598-017-04141-6 (PMC5482821; doi:10.1038/s41598-017-04141-6)

**The Microbiome of Endophytic, Wood Colonizing Bacteria from Pine Trees as Affected by Pine Wilt Disease**

**Additional Supporting Information**

Diogo Neves Proença^1^, Romeu Francisco^1^, Susanne Kublik^2^, Anne Schöler^2^, Gisle Vestergaard^2^, Michael Schloter^2*^ and Paula V. Morais^1,3*^

^1^CEMMPRE, University of Coimbra, Coimbra, Portugal

^2^Research Unit Environmental Genomics, Helmholtz Zentrum München, Munich, Germany

^3^Department of Life Sciences, University of Coimbra, Coimbra, Portugal

Running title: Endophytic microbiome of Pine Trees

Keywords: Endophytes, bacteria, pine wilt disease, amplicon sequencing

^*^authors contributed equally

**Corresponding authors:**

Michael Schloter, email: [schloter@helmholtz-muenchen.de](mailto:schloter@helmholtz-muenchen.de)

**Table S1. Characteristics of pine tree samples collected at Malhada (M) and Avô (A)**

| **Sampling site** | **Sampled trees** | **Tree species** | **DBH^*^ (cm)** | **Localization** | **PWD symptom class^#^** | ***Bursaphelenchus xylophilus*** | **Bacterial isolates** |
| --- | --- | --- | --- | --- | --- | --- | --- |
| **M** | M10 | *P. pinaster* | 33.5 | Trunk | s0 | - | - |
|  | M14 | *P. pinaster* | 28.0 | Trunk | sII | - | - |
|  | M23 | *P. pinaster* | 25.0 | Trunk | sI | - | - |
|  | M24T | *P. pinaster* | 28.0 | Trunk | sV | + | + |
|  | M24C | *P. pinaster* | n.d. | Crown | sV | + | + |
|  | M29 | *P. pinaster* | 20.5 | Trunk | sIII | - | + |
|  | M33 | *P. pinaster* | 21.5 | Trunk | sI | - | - |
|  | M36 | *P. pinaster* | 29.5 | Trunk | s0 | - | - |
|  | M47T | *P. pinaster* | 29.0 | Trunk | sV | + | + |
|  | M47C | *P. pinaster* | n.d. | Crown | sV | + | + |
|  | M67T | *P. pinaster* | 14.0 | Trunk | sV | + | + |
|  | M67C | *P. pinaster* | n.d. | Crown | sV | + | + |
|  | M68T | *P. pinaster* | 11.5 | Trunk | sV | - | + |
|  | M68C | *P. pinaster* | n.d. | Crown | sV | - | + |
|  | M70 | *P. pinaster* | 19,5 | Trunk | s0 | - | - |
|  | M72T | *P. pinaster* | 8.5 | Trunk | sV | - | + |
|  | M72C | *P. pinaster* | n.d. | Crown | sV | - | + |
|  | M73T | *P. pinaster* | 8.5 | Trunk | sIV | - | + |
|  | M73C | *P. pinaster* | n.d. | Crown | sIV | - | + |
|  | M74 | *P. pinaster* | 17.0 | Trunk | sIV | - | - |
|  | M75 | *P. pinaster* | 20.5 | Trunk | s0 | - | - |
|  | M76 | *P. pinaster* | 22.5 | Trunk | s0 | - | - |
|  | M78 | *P. pinaster* | 12.0 | Trunk | sI | - | - |
|  | M94 | *P. pinaster* | 28.5 | Trunk | s0 | - | - |
| **A** | A4 | *P. pinaster* | 30.5 | Trunk | sI | - | - |
|  | A6 | *P. pinaster* | 28.0 | Trunk | sII | - | + |
|  | A9 | *P. pinaster* | 24.5 | Trunk | s0 | - | + |
|  | A12T | *P. pinaster* | 27.0 | Trunk | sIII | + | + |
|  | A12C | *P. pinaster* | n.d. | Crown | sIII | + | + |
|  | A19 | *P. pinaster* | 22.5 | Trunk | s0 | - | - |
|  | A24 | *P. pinaster* | 24.5 | Trunk | s0 | - | + |
|  | A25T | *P. pinaster* | 18.5 | Trunk | sIII | + | + |
|  | A25C | *P. pinaster* | n.d. | Crown | sIII | + | - |
|  | A36T | *P. pinaster* | 26.0 | Trunk | sIV | + | - |
|  | A36C | *P. pinaster* | n.d. | Crown | sIV | + | + |
|  | A37T | *P. pinaster* | 24.5 | Trunk | sV | + | + |
|  | A37C | *P. pinaster* | n.d. | Crown | sV | + | + |
|  | A41T | *P. pinaster* | 24.0 | Trunk | sIII | + | + |
|  | A41C | *P. pinaster* | n.d. | Crown | sIII | + | + |
|  | A42 | *P. pinaster* | 23.5 | Trunk | s0 | - | - |
|  | A45 | *P. pinaster* | 42.5 | Trunk | sI | - | + |
|  | A52T | *P. pinaster* | 7.5 | Trunk | sV | + | + |
|  | A52C | *P. pinaster* | n.d. | Crown | sV | - | + |
|  | A60T | *P. pinaster* | 34.0 | Trunk | sIII | + | + |
|  | A60C | *P. pinaster* | n.d. | Crown | sIII | - | - |
|  | A64T | *P. pinaster* | 18.0 | Trunk | sIV | - | - |
|  | A64C | *P. pinaster* | n.d. | Crown | sIV | - | - |
|  | A72T | *P. pinaster* | 30.0 | Trunk | sII | + | - |
|  | A72C | *P. pinaster* | n.d. | Crown | sII | + | - |
|  | A87 | *P. pinaster* | 30.0 | Trunk | s0 | - | - |
|  | A87s | *P. pinaster* | n.d. | Trunk | s0 | - | + |
|  | A88T | *P. pinaster* | 23.0 | Trunk | sIII | + | + |
|  | A88C | *P. pinaster* | n.d. | Crown | sIII | + | + |
|  | A96T | *P. pinaster* | 28.5 | Trunk | sII | + | + |
|  | A96C | *P. pinaster* | n.d. | Crown | sII | + | + |
|  | A103T | *P. pinaster* | 46.0 | Trunk | sV | + | + |
|  | A103C | *P. pinaster* | n.d. | Crown | sV | - | - |
|  | B20T | *P. pinaster* | 10.8 | Trunk | sV | - | - |
|  | B20C | *P. pinaster* | n.d. | Crown | sV | + | - |
|  | B24T | *P. pinaster* | 12.5 | Trunk | s0 | - | - |
|  | B24C | *P. pinaster* | n.d. | Crown | s0 | - | - |
|  | B23T | *P. pinaster* | 12.2 | Trunk | sV | + | + |
|  | B23C | *P. pinaster* | n.d. | Crown | sV | + | + |
|  | B29T | *P. pinaster* | 11.8 | Trunk | sV | + | + |
|  | B29C | *P. pinaster* | n.d. | Crown | sV | + | + |

^*^DBH - Diameter Breast Heigh.

^#^According to Proença et al. (2010)

n.d. not determined

**Table S2.** Diversity of bacterial microbiomes from *P. pinaster* determined by barcoding and fingerprinting of the 16S rRNA gene as well as isolation

|  | **Barcoding (Seq)** | | | **Fingerprinting** | | | | | **Isolates** | | | |
| --- | --- | --- | --- | --- | --- | --- | --- | --- | --- | --- | --- | --- |
| **Samples** | **Shannon** | **Simpson_1-D** | **Chao1** | **Shannon** | **Simpson_1-D** | | **Chao1** | | **Shannon** | | **Simpson_1-D** | **Chao1** |
| Malhada-s0 | 4.81 | 0.97 | 976.0 | 3.13 | 0.95 | 44.2 | | ND | | ND | | ND |
| Malhada-sI | 3.95 | 0.87 | 926.2 | 3.31 | 0.96 | 39.5 | | ND | | ND | | ND |
| Malhada-sII | 3.74 | 0.84 | 1,213.1 | 2.77 | 0.94 | 136.0 | | ND | | ND | | ND |
| Malhada-sIII | 4.13 | 0.93 | 1,578.4 | 2.30 | 0.90 | 55.0 | | 1.93 | | 0.79 | | 20.5 |
| Malhada-sIV | 5.77 | 0.99 | 3,225.3 | 2.66 | 0.92 | 21.3 | | 1.73 | | 0.77 | | 13.0 |
| Malhada-sV | 5.53 | 0.99 | 2,515.6 | 3.27 | 0.96 | 34.5 | | 2.77 | | 0.91 | | 40.75 |
| Avô-s0 | 4.64 | 0.97 | 978.5 | 2.71 | 0.93 | 21.0 | | 0.95 | | 0.56 | | 4.0 |
| Avô-sI | 3.32 | 0.90 | 485.1 | 2.34 | 0.90 | 15.2 | | 0.00 | | 0.00 | | 1.0 |
| Avô-sII | 3.68 | 0.93 | 984.4 | 2.62 | 0.92 | 15.3 | | 1.38 | | 0.70 | | 5.5 |
| Avô-sIII | 1.18 | 0.36 | 451.1 | 2.79 | 0.93 | 19.3 | | 2.11 | | 0.85 | | 13.5 |
| Avô-sIV | 4.18 | 0.95 | 1,223.9 | 2.78 | 0.94 | 17.0 | | 0.60 | | 0.31 | | 4.0 |
| Avô-sV | 5.12 | 0.98 | 2,314.6 | 2.92 | 0.94 | 23.8 | | 2.76 | | 0.91 | | 30.2 |

**Table S3.** Number of reads and OTUs after chloroplast sequences were removed using QIIME and the Greengenes database, from 12 samples covering trees of six different symptomatic physiological classes from the two sampling areas.

| **Samples** | **Total read pairs** | **Filtered and trimmed bp** | **% Chloroplast** | **Final read pairs** | **OTUs** |
| --- | --- | --- | --- | --- | --- |
| Malhada-s0 | 86,321 | 23,190,233 | 62 | 24,069 | 976 |
| Malhada-sI | 73,851 | 20,378,357 | 57 | 24,555 | 926 |
| Malhada-sII | 97,406 | 27,711,094 | 42 | 45,931 | 1,072 |
| Malhada-sIII | 96,733 | 20,970,129 | 0 | 55,504 | 1,267 |
| Malhada-sIV | 132,771 | 34,430,984 | 13 | 51,121 | 2,260 |
| Malhada-sV | 89,738 | 20,724,569 | 2 | 51,121 | 2,058 |
| Avô-s0 | 60,735 | 18,768,866 | 14 | 46,191 | 870 |
| Avô-sI | 55,806 | 16,893,239 | 18 | 39,851 | 454 |
| Avô-sII | 161,477 | 50,918,059 | 27 | 107,047 | 686 |
| Avô-sIII | 112,258 | 36,318,184 | 1 | 101,778 | 981 |
| Avô-sIV | 220,607 | 68,916,006 | 3 | 192,507 | 812 |
| Avô-sV | 208,182 | 62,562,059 | 1 | 176,403 | 1,410 |

**Table S4.** Phenotypic characterization of isolates from genera contributing more than 1% to the endophytic microbiome. Strains were tested for phosphate (PO4) and zinc (Zn) solubilization, siderophores (Sid) production, proteolytic (Prot) and lipolytic (T20, T40, T60 and T80) activity, hydrolysis of cellulose (CMC) and chitin degradation.

| **Genera** | **PO4** | **Zn** | **Sid** | **Prot** | **T20** | **T40** | **T60** | **T80** | **CMC** | **Chitin** |
| --- | --- | --- | --- | --- | --- | --- | --- | --- | --- | --- |
| *Bacillus* | 0.0 | 44.4 | 55.6 | 66.7 | 22.2 | 33.3 | 33.3 | 0.0 | 44.4 | 0.0 |
| *Burkholderia* | 21.1 | 10.5 | 63.2 | 36.8 | 47.4 | 52.6 | 52.6 | 21.1 | 31.6 | 0.0 |
| *Chitinophaga* | 0.0 | 0.0 | 100.0 | 0.0 | 0.0 | 0.0 | 0.0 | 0.0 | 100.0 | 0.0 |
| *Dyella* | 0.0 | 16.7 | 33.3 | 50.0 | 100.0 | 83.3 | 33.3 | 0.0 | 0.0 | 0.0 |
| *Erwinia* | 72.4 | 69.0 | 89.7 | 17.2 | 17.2 | 24.1 | 55.2 | 3.4 | 17.2 | 0.0 |
| *Ewingella* | 0.0 | 100.0 | 100.0 | 0.0 | 100.0 | 100.0 | 100.0 | 0.0 | 100.0 | 0.0 |
| *Flavobacterium* | 0.0 | 0.0 | 100.0 | 100.0 | 100.0 | 100.0 | 0.0 | 0.0 | 0.0 | 0.0 |
| *Luteibacter* | 0.0 | 0.0 | 50.0 | 100.0 | 90.0 | 40.0 | 40.0 | 0.0 | 10.0 | 0.0 |
| *Novosphingobium* | 0.0 | 20.0 | 20.0 | 20.0 | 0.0 | 20.0 | 60.0 | 0.0 | 80.0 | 0.0 |
| *Pseudomonas* | 0.0 | 66.7 | 55.6 | 22.2 | 22.2 | 11.1 | 55.6 | 0.0 | 22.2 | 0.0 |
| *Pseudoxanthomonas* | 67.5 | 75.0 | 77.5 | 45.0 | 72.5 | 70.0 | 62.5 | 30.0 | 10.0 | 0.0 |
| *Serratia* | 0.0 | 57.1 | 71.4 | 42.9 | 42.9 | 0.0 | 85.7 | 42.9 | 0.0 | 42.9 |
| *Sphingomonas* | 19.4 | 11.1 | 27.8 | 22.2 | 33.3 | 33.3 | 44.4 | 0.0 | 25.0 | 0.0 |
| *Staphylococcus* | 0.0 | 0.0 | 100.0 | 0.0 | 100.0 | 50.0 | 0.0 | 0.0 | 50.0 | 0.0 |
| *Stenotrophomonas* | 0.0 | 0.0 | 100.0 | 100.0 | 100.0 | 100.0 | 0.0 | 0.0 | 0.0 | 50.0 |
| *Streptococcus* | 0.0 | 0.0 | 0.0 | 0.0 | 0.0 | 100.0 | 0.0 | 0.0 | 0.0 | 0.0 |
| *Terriglobus* | 0.0 | 0.0 | 0.0 | 0.0 | 50.0 | 50.0 | 50.0 | 0.0 | 0.0 | 0.0 |
| *Variovorax* | 0.0 | 0.0 | 25.0 | 0.0 | 0.0 | 50.0 | 50.0 | 0.0 | 25.0 | 0.0 |
| TOTAL | 31.9 | 40.0 | 60.0 | 35.7 | 45.9 | 43.8 | 51.4 | 10.8 | 21.6 | 2.2 |

**Figure S1.** Rarefaction analysis curves of endophytic, wood colonizing bacterial communities of pine tree samples in six symptomatic conditions of Pine Wilt Disease from site M and site A. Sequences were clustered into OTUs using a sequence identity threshold of 97%. Rarefaction curves indicate sufficient sequencing depth to assess OTU diversity of early stages of disease (s0-sII) samples, while a large increase of OTU diversity at later stages (sIV and sV) was observed.


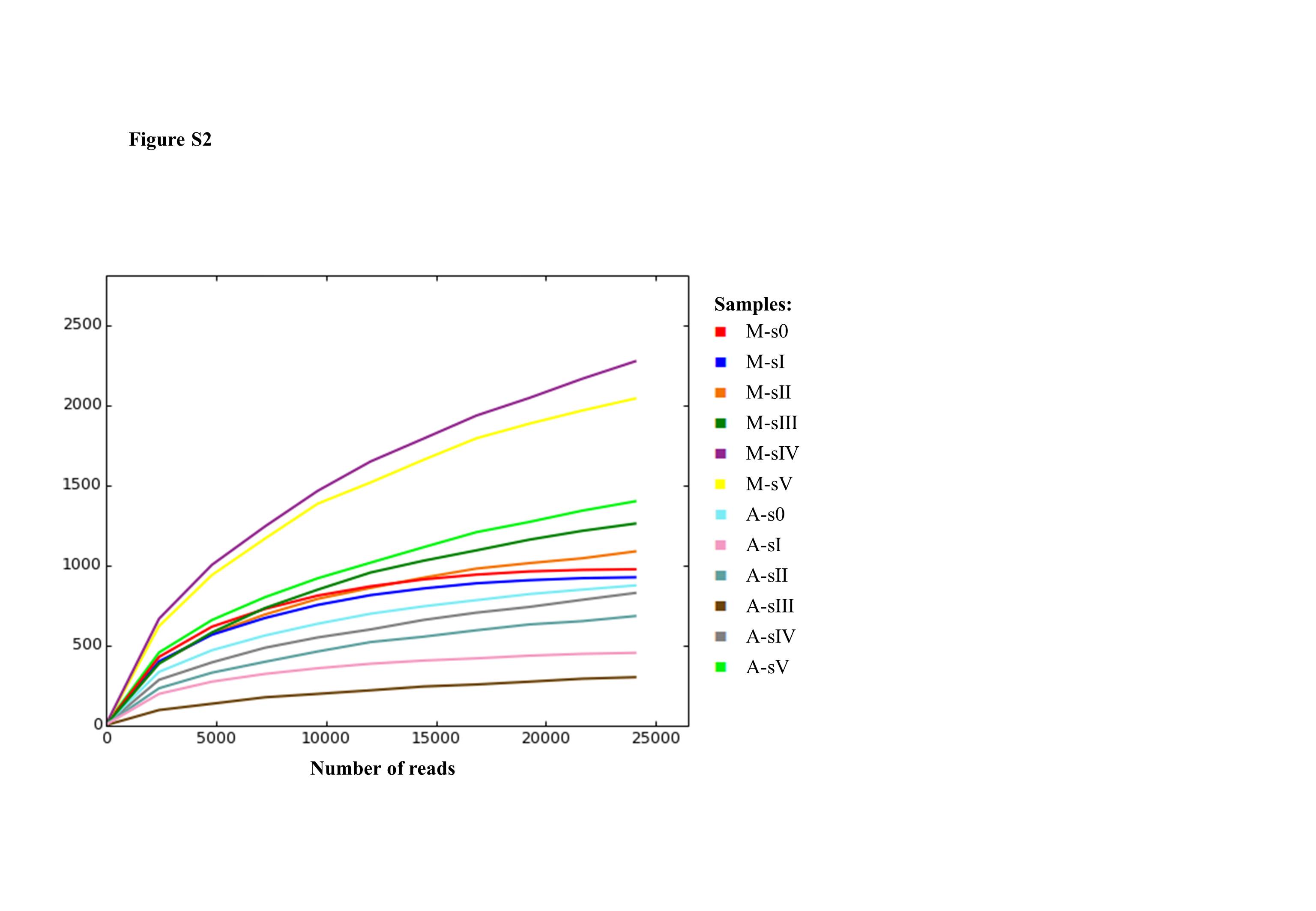


**Figure S2.** β-Diversity of endophytic, wood colonizing bacterial communities of *Pinus pinaster* samples affected by Pine Wilt Disease from site M and site A. Bray-Curtis dissimilarity principal coordinates analysis (PCoA) was used to generate ordination of beta-diversity in three dimensions. Principal coordinates 1, 2 and 3 (PC1, PC2 and PC3) explain 43.83%, 21.91% and 12.79% of the variance in Bray-Curtis dissimilarity, respectively. Increasing distance in three-dimensional space represents increasingly dissimilar community structures and samples are colored according to the symptomatic disease conditions in the legend.

**
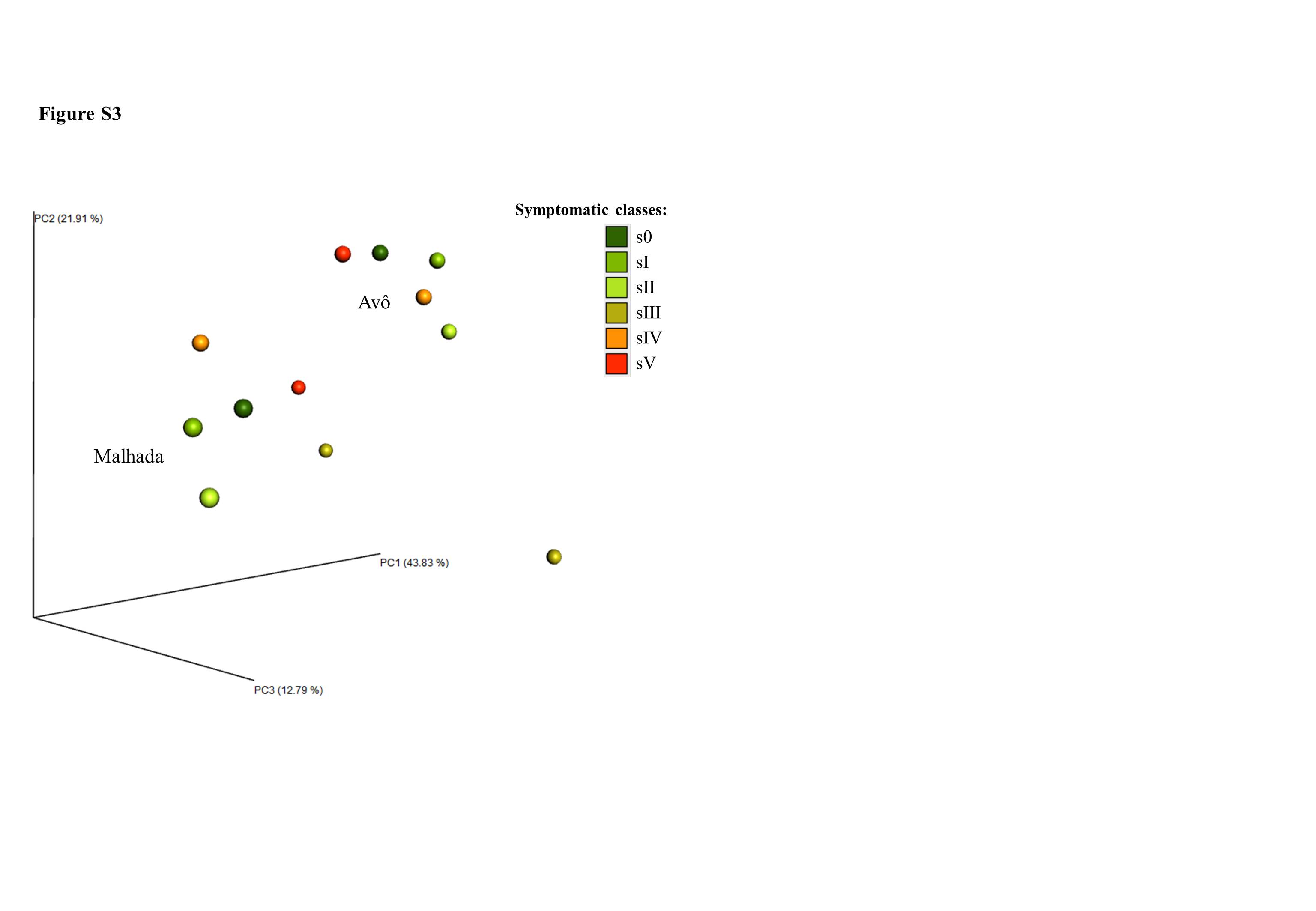
**

**Figure S3.** Phenotypic characterization of endophytic, wood colonizing bacteria obtained by cultivation based approaches from pine trees of the two study sites A and M (symptom classes 3 – 5).

**
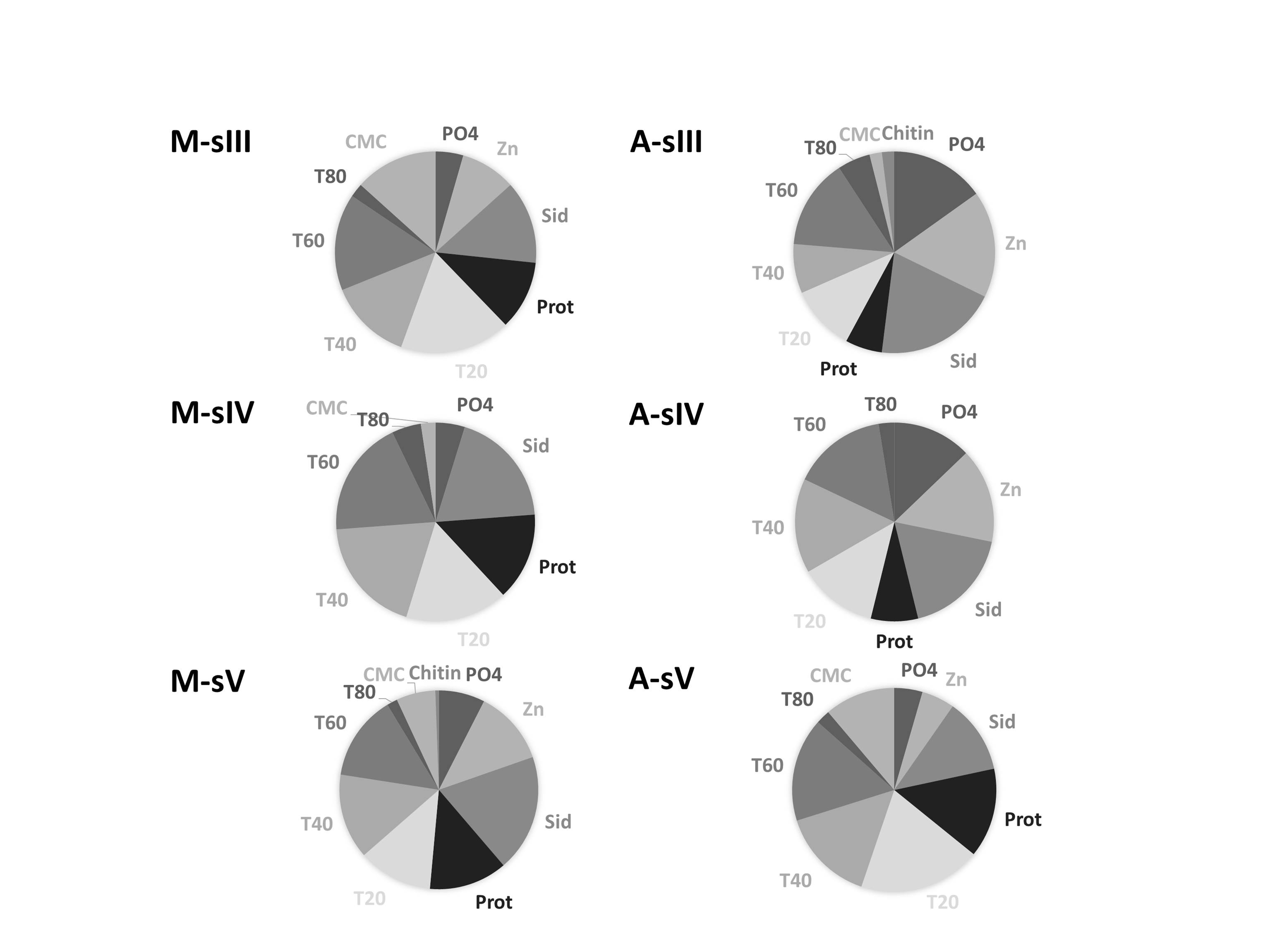
**

**Figure S4.** Phylogenetic dendrogram based on a comparison of the 16S rRNA gene sequences of endophytic bacteria from this study (bacterial isolates - dark orange; representative OTU ID - light orange) and bacteria carried by PWN obtained from different countries (Portugal - green; USA – blue; China – red). The tree was created using the neighbor-joining method. The numbers on the tree indicate the percentages of bootstrap sampling, derived from 1,000 replications; values below 50% are not shown. Symbol (
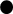
 ) indicates node branches conserved when the tree was reconstructed using the maximum parsimony algorithm (RAxML). The isolates characterized in this study are indicated in dark and light orange. Scale bar, 2 inferred nucleotide substitution per 100 nucleotides.


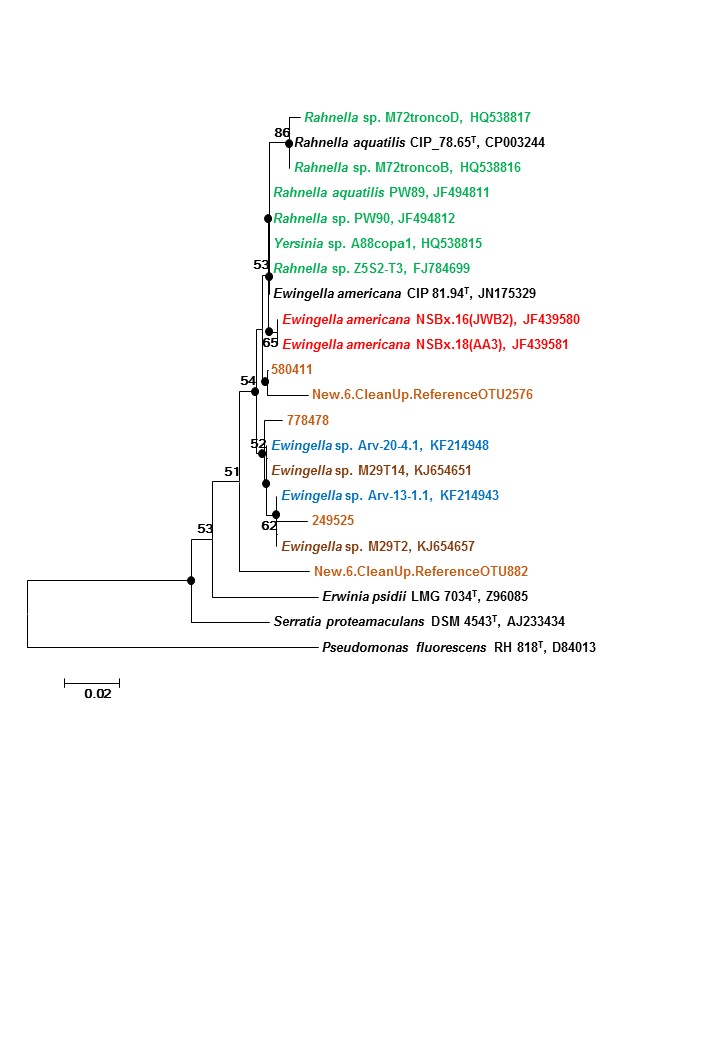

Supplement: Supplementary file 1 — Supplement [file 41598_2017_4141_MOESM1_ESM.doc]
